# Co-Circulation of Multiple Hemorrhagic Fever Diseases with Distinct Clinical Characteristics in Dandong, China

**DOI:** 10.1371/journal.pone.0089896

**Published:** 2014-02-27

**Authors:** Zhi-Hai Chen, Xin-Cheng Qin, Rui Song, Yi Shen, Xiao-Ping Chen, Wen Wang, Yong-Xiang Zhao, Jing-Shan Zhang, Jin-Rong He, Ming-Hui Li, Xue-Hua Zhao, De-Wei Liu, Xiao-Kang Fu, Di Tian, Xing-Wang Li, Jianguo Xu, Alexander Plyusnin, Edward C. Holmes, Yong-Zhen Zhang

**Affiliations:** 1 Department of Infectious Diseases, Beijing Ditan Hospital, Capital Medical University, Beijing, China; 2 State Key Laboratory for Infectious Disease Prevention and Control, Collaborative Innovation Center for Diagnosis and Treatment of Infectious Diseases, Department of Zoonoses, National Institute for Communicable Disease Control and Prevention, Chinese Center for Disease Control and Prevention, Beijing, China; 3 Department of Infectious Diseases, Dandong Infectious Hospital, Dandong, Liaoning Province, China; 4 Department of Virology, Haartman Institute, University of Helsinki, Finland; 5 Marie Bashir Institute for Infectious Diseases and Biosecurity, School of Biological Sciences and Sydney Medical School, The University of Sydney, Sydney, Australia; Tulane School of Public Health and Tropical Medicine, United States of America

## Abstract

Hemorrhagic fevers (HF) caused by viruses and bacteria are a major public health problem in China and characterized by variable clinical manifestations, such that it is often difficult to achieve accurate diagnosis and treatment. The causes of HF in 85 patients admitted to Dandong hospital, China, between 2011–2012 were determined by serological and PCR tests. Of these, 34 patients were diagnosed with Huaiyangshan hemorrhagic fever (HYSHF), 34 with Hemorrhagic Fever with Renal Syndrome (HFRS), one with murine typhus, and one with scrub typhus. Etiologic agents could not be determined in the 15 remaining patients. Phylogenetic analyses of recovered bacterial and viral sequences revealed that the causative infectious agents were closely related to those described in other geographical regions. As these diseases have no distinctive clinical features in their early stage, only 13 patients were initially accurately diagnosed. The distinctive clinical features of HFRS and HYSHF developed during disease progression. Enlarged lymph nodes, cough, sputum, and diarrhea were more common in HYSHF patients, while more HFRS cases presented with headache, sore throat, oliguria, percussion pain kidney area, and petechiae. Additionally, HYSHF patients displayed significantly lower levels of white blood cells (WBC), higher levels of creations kinase (CK) and alanine aminotransferase (ALT), while HFRS patients presented with an elevation of blood urea nitrogen (BUN) and creatinine (CREA). These clinical features will assist in the accurate diagnosis of both HYSHF and HFRS. Overall, our data reveal the complexity of pathogens causing HFs in a single Chinese hospital, and highlight the need for accurate early diagnosis and a better understanding of their distinctive clinical features.

## Introduction

Diseases known by the collective term “Hemorrhagic fever (HF)” are important human infections often with high mortality, and with such notable examples as dengue hemorrhagic fever, Ebola hemorrhagic fever, and Hemorrhagic Fever with Renal Syndrome (HFRS). Clinically, these diseases are often characterized by severe fever and coagulopathy. Some HFs display a clear set of clinical stages, such as HFRS with distinct renal symptoms [1]. Etiologic studies have identified several viruses as causative agents, especially RNA viruses from the families *Arenaviridae*, *Bunyaviridae*, *Filoviridae*, and *Flaviviridae* [2], [3]. *Rickettsiales* bacteria, such as *Rickettsia typhi*, *Orientia tsutsugamushi*, and *Anaplasma phagocytophilums*, can also cause HF [4]. However, the clinical manifestations and severity of HFs vary significantly, from extremely high fatality rates in Ebola hemorrhagic fever to asymptomatic in many dengue viral infections [3], and severe fever and coagulopathy are also present in diseases such as leptospirosis and malaria. To date, commercial kits are not available for the diagnosis of most HFs. Hence, it is difficult to accurately diagnose HF diseases from clinical presentation alone. As a consequence it is clear that a better understanding of the clinical characteristics of HF diseases caused by different pathogens is central to improved diagnostics and treatment.

Several pathogens are known to cause HF in China. Indeed, China is one of the most important endemic areas of HFRS (caused by hantaviruses) worldwide, with more than 1.5 million reported cases and 44,475 deaths during 1970–2007 [5]. More than 10 hantaviruses have been identified in bats, rodent, and shrews sampled from China [5]–[8]. HFRS cases are mainly caused by Hantaan virus (HTNV) and Seoul virus (SEOV) transmitted by rodents [5]. Recently, Gou virus (GOUV), also rodent-borne, was found to cause HFRS [9]. HFs caused by other viruses (e.g. Crimean-Congo hemorrhagic fever virus (CCHFV), dengue virus) and bacteria (e.g. *Rickettsiales*, *Leptospira*) are also endemic in China [10]–[14]. However, due to the lack of commercial specific diagnostics for most HF pathogens (especially *Rickettsiales* bacteria), many HF cases in China are diagnosed entirely using clinical criteria and hence often misdiagnosed. In addition, other HFs may be caused by as yet unrecognized pathogens.

In 2009, a new HF disease (Huaiyangshan hemorrhagic fever, HYSHF, also known as severe fever with thrombocytopenia syndrome) with a 15% mortality rate was documented in the Huaiyangshan region of China [15]–[17]. The disease was caused by a novel virus (Huaiyangshan virus, HYSV) transmitted by ticks, most closely related to viruses of the genus *Phlebovirus* (family *Bunyaviuridae*) [15]. Although several papers have described the clinical presentation and progression of HYSHF [15]–[19], no studies have compared the clinical features of HYSHF to those of other HFs in China. Recently, a disease with similar symptoms and caused by HYSV-like virus was reported in the USA [20]. Better understanding of the clinical course of HYSHF and other HFs may be helpful in the accurate diagnosis and treatment of these devastating diseases.

Dandong city with a population of around 2,453,000 (including both urban and rural areas) is located in the southern part of Liaoning Province bordering North Korea (Figure S1). Within Liaoning Province Dandong has been severely affected by HFRS [21], although the full range of HFs and causative viruses and bacteria present in the population is unknown. To better understand the HFs in Dandong, with the aim of improving clinical diagnosis and treatment, we analyzed 85 patients admitted to Dandong Infectious Disease Hospital during 2011–2012 suffering from severe fever and/or hemorrhage(s). The etiologic agents, clinical manifestations, symptom distribution, and therapeutic responses of these patients were investigated. Additionally, we compared the differences in clinical features between HYSHF and HFRS, and explored the molecular epidemiology of these pathogens.

## Materials and Methods

### Ethics statement

This study was reviewed and approved by the ethics committees of Dandong Infectious Disease Hospital and Beijing Ditan Hospital. The collection of human serum samples from HF patients was also approved by the ethics committees of Dandong Infectious Disease Hospital and Beijing Ditan Hospital, according to the medical research regulations of the Ministry of Health, China. A signed individual written informed consent was obtained from each of 84 adult patients when their blood samples were collected. For one patient of four years of age written consent was provided by his parents.

### Case definitions and clinical data

We recruited patients for two years (between January 2011 and December 2012) from Dandong Infectious Disease Hospital in Dandong city, Liaoning Province, China. Patients were recruited through review of clinical analysis.

The case definition included any person of any age admitted to hospital with acute onset of fever (>37.5°C), thrombocytopenia (<100×10^9^/L), and/or hemorrhagic manifestations. The cases were primarily diagnosed according to the national clinical and laboratory criteria listed by the Ministry of Health, China for HYSHF, HFRS, murine typhus, scrub typhus, and other related diseases (Table S1). Generally, physicians made their primary diagnoses according to their clinical experience when patients were admitted into the hospital. They made their clinical diagnoses combining clinic presentations with biochemical parameters, and the final diagnoses for HFRS based on laboratory tests when the related specific reagents were available.

Laboratory diagnosis was based on the presence of specific immunoglobulin M (IgM) antibodies or the four fold increase of IgG antibody titer, or following the isolation and characterization of specific viral and bacterial genome sequences (see below) in the Department of Zoonoses of the National Institute for Communicable Disease Control and Prevention, China CDC in Beijing.

Demographic and clinical data, medication/treatment history, and the description of clinical course including the time before medical attention, clinical symptoms such as fever, headache, myalgia, nausea, malaise, hemorrhage, pulmonary and hepatic dysfunction, renal dysfunction, and neurological involvement, were collected and evaluated.

### Laboratory tests

During admission, blood samples were collected from each patient at day 1 and at follow-up during admission. Blood cell counts and differentials were routinely examined by the Clinical Diagnosis Laboratory of the Dandong Infectious Disease Hospital. Blood cultures for anaerobes and aerobes were repeated three times for the detection of bacterial agents. The levels of plasma alanine transaminase (ALT), aspartate transaminase (AST), lactate dehydrogenase (LDH), and creatine phosphatase (CK) in individual patients, thrombin time (TT), and other biochemical parameters were first evaluated by the Clinical Diagnosis Laboratory of the Dandong Infectious Disease Hospital and then confirmed in the Biochemical Laboratory of Beijing Ditan Hospital.

### Serologic analysis

Blood samples collected from suspected HFRS patients were first tested in the Dandong Infectious Disease Hospital for hantavirus (HV)-specific antibodies by indirect immunofluorescent assay (IFA) as described previously [22]. All sera from patients were tested in the Department of Zoonoses of the National Institute for Communicable Disease Control and Prevention, China CDC in Beijing for specific IgM and IgG antibodies against HYSV, HV (including HTNV and SEOV), *R. typhi, R. prowazekii, O. tsutsugamushi, A. phagocytophilum*, and *E*. *chaffeensis* by IFA as described previously [12], [15], [22]. HYSV, HTNV, and SEOV infected cells were spread onto slides, air-dried, and fixed with acetone. These slide antigens were used to detect HYSV and HV, while those for the detection of *R. typhi, R. prowazekii, O. tsutsugamushi, A. phagocytophilum*, and *E. chaffeensis* were purchased either from Focus Diagnostics (Cypress, CA, USA) or Fuller Laboratories (Fullerton, CA, USA). For detection of *Leptospira* bacteria, microscopic agglutination tests (MAT) were performed using a battery of anti-serum against the Chinese reference strains belonging to 15 serovars in 15 serogroups [23].

### PCR and sequencing

RNA and DNA were extracted from all blood samples using the QIAamp MinElute Virus Spin Kit (Qiagen, Valencia, CA, USA). For the detection of HYSV, HV, Kysanur forest diseases virus, Omsk hemorrhagic fever virus, Crimean-Congo hemorrhagic fever virus, viral RNA in blood samples from individual patients was recovered by reverse transcription (RT)-PCR as described previously [15], [22], [24]–[26].

Rickettsiale DNA was detected by PCR as described previously by using primers EHR16SD and rp1588 (5′-ACRGCTACCTTGTTACGACT-3′) for the initial PCR, and primers EHR16SD and 16SR871 (5′-AGCACTCATCGTTTACAGCG-3′) for the second round of amplification [27], [28], which yielded the 345 nt fragment of the 16S rRNA gene (16S rDNA) of *Rickettsiales* bacteria. *Leptospira* DNA was also detected by PCR as described previously [29].

PCR products were gel-purified and subjected to sequencing with the 3730 DNA Sequence Analyzer (Applied Biosystems, USA).

### Phylogenetic analysis

All viral and bacterial sequences were aligned and edited with the ClustalW program (DNASTAR, Inc., Madison, WI). Phylogenetic trees were estimated using the Bayesian method implemented in MrBayes 3.1 and the Maximum likelihood (ML) method available in the RAxML Blackbox webserver [30], [31], under the best-fit GTR+I+Γ_4_ model of nucleotide substitution established using jModeltest [32]. For the Bayesian analysis, we used three hot and one cold Markov chain Monte Carlo (MCMC) chain, sampling every 100 generations and with a 25% burnin. The Effective Sample Size (ESS) of all parameters was larger than 200, suggesting that parameter convergence had occurred. Topological support was evaluated by node posterior probabilities for the Bayesian trees and bootstrap support values for the ML trees, respectively.

In addition to the sequences recovered in this study, we used comparison viral and bacterial sequences retrieved from GenBank (www.ncbi.nlm.nih.gov/Genbank) for phylogenetic analyses (Table S2).

### Statistical analysis

Statistical analyses were performed using SPSS (version 16). The comparison of HYSHF and HFRS laboratory parameters was undertaken using a Chi-square test or Fisher's exact test, with a p-value <0.05 considered statistically significant.

## Results

### Demographic characteristics and clinical epidemiology

The demographic characteristics of the 85 HF patients are described in Table 1. Notably, HYSHF cases appeared from July to October, HFRS cases were observed in the winter, and undetermined infections were observed in summer, autumn, and winter (Figure S2). All cases occurred in rural areas and all patients were farm workers. The patients included 59 males and 26 females, with ages ranging from 12 to 89 years (median of 54 years).

**Table 1:** Demographic characteristics of 85 patients with HF.

Variable	HYSHF (%)	HFRS (%)	Murine typhus	Scrub typhus	Undetermined infection (%)
Number	34	34	1	1	15
Age (years)					
Median	61	51.5	63	89	42
Range	41–86	32–75			12–80
Age groups (years)					
<20	0	0			1 (6.7)
21–30	0	0			2 (13.3)
31–40	0	2 (5.9)			2 (13.3)
41–50	11 (32.4)	15 (44.1)			4 (26.7)
51–60	6 (17.6)	8 (23.5)			4 (26.7)
>60	17 (50.0)	9 (26.5)	1	1	2 (13.3)
Gender					
Male	17 (50.0)	28 (82.4)	1		13 (86.7)
Female	17 (50.0)	6 (17.6)		1	2 (13.3)

Abbreviations: HF, hemorrhagic fever; HYSHF, Huaiyangshan hemorrhagic fever; HFRS, hemorrhagic fever with renal syndrome.

### Laboratory diagnoses and clinical diagnoses

During the period January 2011 to December 2012 a total of 85 suspected patients with symptoms compatible with HF were prospectively investigated at Dandong Infectious Disease Hospital. According to serological tests and genetic analyses, 34 patients suffered from HYSHF, 34 from HFRS, one from murine typhus, and one from scrub typhus, which was the first human case reported in northeastern China (Table 2).

**Table 2:** Detection of pathogen-specific antibodies by indirect immunofluorescence and specific RNA/DNA by RT-PCR in serum samples.

Pathogens	Antibodies			RT-PCR or PCR	Laboratory confirmed infections
	IgM	IgG	Four fold increase in IgG titers		
Huaiyangshan virus	18	26	23	30	34
HTNV	15	24	22	24	33
SEOV*	0	1	0	0	1
*Rickettsia typhi*	0	1	1	0	1
*Orentia tsutsugamushi*	0	1	0	1	1
*Ehrlichia chaffeensis*	0	0	0	0	0
*Anaplasma phagocytophilum*	0	0	0	0	0
*Leptospira*	0	0	0	0	0
Kysanur forest diseases virus	-	-	-	0	0
Omsk hemorrhagic fever virus	-	-	-	0	0
CCHFV	-	-	-	0	0
Undetermined	-	-	-	-	15

Abbreviations: HTNV, Hantaan virus; SEOV, Seoul virus; CCHFV, Crimean-Congo hemorrhagic fever virus.

-: Not available.

*: As the patient (without receiving HV vaccination) had a high level of IgG antibodies (1∶320) against SEOV it was diagnosed as SEOV infection even though it does not meet our defined case criteria (i.e. four-fold rise of IgG not demonstrated, IgM negative, PCR negative).

Although only seven HFRS cases were recognized by clinical symptoms at an early stage (Table 3), 31 HFRS cases (91.18%) were accurately diagnosed at the point of discharge based on laboratory tests performed at the Dandong Infectious Disease Hospital (as the reagents for HTNV and SEOV diagnostics are now available in China). However, as the diagnostic reagents for HYSV and *Rickettsiales* agents were not available at Dandong Infectious Disease Hospital, only six (17.6%) HYSHF cases were accurately diagnosed according to clinical criteria (Table 3), with the remaining 28 cases misdiagnosed as either HFRS or Rickettsial disease, or referred to as “undetermined infections”. Moreover, murine typhus and scrub typhus were misdiagnosed as sepsis and HFRS, respectively. Fourteen of 15 undetermined infections (93.33%), which could not be identified by the laboratory tests, were clinically misdiagnosed as HYSHF, HFRS, non-specified viral infection, or typhoid.

**Table 3:** Comparison of clinical and laboratory diagnoses of patients with HF.

Laboratory diagnoses	Clinical diagnoses						
	HYSHF	HFRS	Viral infection	Rickettsial diseases	Typhoid	Sepsis	Undetermined infection
HYSHF (n = 34)	6	3	9	11			5
HFRS (n = 34)		7					27
Murine typhus (n = 1)						1	
Scrub typhus (n = 1)		1					
Undetermined infection (n = 15)	2	8	2		2		1

Abbreviations: HF, hemorrhagic fever; HYSHF, Huaiyangshan hemorrhagic fever; HFRS, hemorrhagic fever with renal syndrome.

Clinical diagnoses were made based on clinical findings and suggestive biochemical parameters according to Ministry of Health guidelines.

Laboratory diagnoses were made based on bacterial culture, serological and molecular tests.

### Phylogenetic analyses of viral and bacterial sequences

Both HYSV and HV genome sequences were successfully amplified from patient serum samples. These sequences were designated as ‘DandongHu’-sequences (Table S2). In addition, 16S rDNA sequences were recovered from the *O. tsutsugamushi*-positive serum sample. Unfortunately, our attempts to amplify the genome sequences of other pathogens were unsuccessful (Table 2).

**Figure 1:**
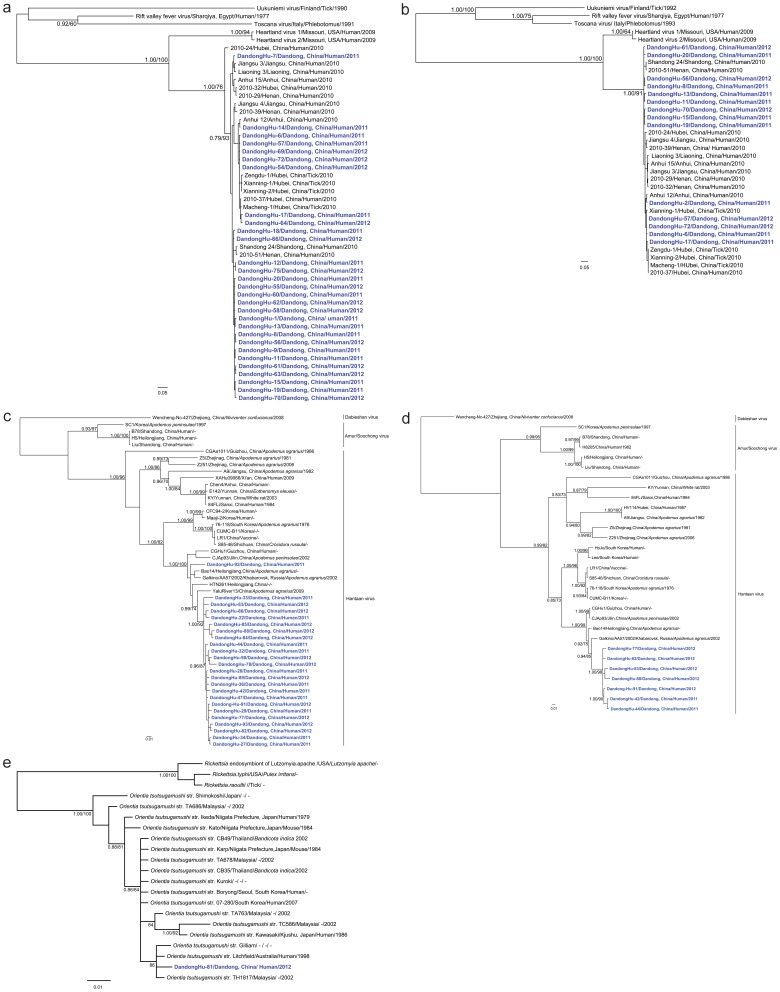
Phylogenetic relationships among Huaiyangshan virus (HYSV) and hantavirus variants found in humans within and outside of China. The Bayesian trees were inferred using the (a) partial S and (b) partial L segment sequences of HYSV, the (c) partial S and (d) partial M sequences of HVs, and (e) the 16S DNA gene of *Rickettsiales*. The numbers above or below branches indicate posterior node probabilities and bootstrap support values. Posterior node probabilities over 0.7 or a 70% bootstrap value were as considered as strong support for a specific node. The blue color represents HYSV, HV, or *O. tsutsugamushi* sequences recovered from human samples collected in Dandong. Scale bars indicate the number of nucleotide substitutions per site. For more detailed information on the viruses and bacteria used in this study see Table S2.

Partial S or L segment sequences were recovered from 30 serum samples collected from HYSHF patients. Genetic analysis showed that these sequences were very closely related to each other (95.9–100% and 96.8–100% sequence identity in the partial S and L segment sequences, respectively), and some were closely related to those recovered from humans located in the Huaiyangshan mountain region of China in 2010 (94.2–99.5% and 96.0–99.5%) [15], [33]. Indeed, in the S phylogenic tree, the sequences from Dandong were divided into three lineages that clustered with sequences recovered from humans and ticks in other parts of China (Figures 1a and 1b) [15], [17], [18], [33].

Complete (or partial) S or M segment sequences were amplified from 24 serum samples from HFRS patients. Interestingly, the majority of these sequences were very closely related to each other, with 93.6–100% identities in the S segment and 96.7–99.5% identities in the M segment, and forming a monophyletic group in phylogenetic trees (Figures 1c and 1d). These sequences were also closely related to those (YaluRiver13, HQ611981) recovered from *A. agrarius* sampled in the neighboring region of Dandong. In addition, the sequences (Dandong-Hu-92) formed another group in the S and M trees, and shared a close relationship with those (CGHu1) isolated from an HFRS patient (34) and from *Apodemus* mice (Bao14, CJAp93) (35, 36). In addition, all these sequences were located close to those recovered from humans and *Apodemus* mice from the northeastern China and Far East Asia (86.8–96.6 and 87.3–95% identity) [34]–[38], some of which (Hojo, Lee, Maaji-2) were associated with severe HFRS cases [1].

Finally, the 16S rDNA sequences recovered from the single scrub typhus patient in this study were closely related to the corresponding sequences of strains found both inside and outside of China, with 97–100% nucleotide identities (Figure 1e) [39], [40].

### Clinical characteristics of HYSHF, HFRS and infections caused by unknown agent(s)

Disease began abruptly with high fever, headache and myalgia, nausea, vomiting and diarrhea, along with a variety of other nonspecific signs and symptoms (Table 4). However, within a few days some patients exhibited coagulation defects and mild hemorrhagic symptoms, such as skin rash, petechiae, and hematuria. Notably, enlarged lymph nodes were only observed in the HYSHF cases (44.12%).

**Table 4:** Clinical characteristics of patients with HF.

Clinical feature	HYSHF (n = 34)	HFRS (n = 34)	Murine typhus (n = 1)	Scrub typhus (n = 1)	Undetermined infections (n = 15)
Fever	34 (100.0)	34 (100.0)	+	+	15 (100.0)
Headache	20 (58.82)	30 (88.24)*	+	+	12 (80.00)
Dizziness	17 (50.00)	12 (35.29)	−	−	5 (33.33)
Chills	12 (35.29)	11 (32.35)	−	+	8 (53.33)
Myalgia	20 (58.82)	27 (79.41)	−	+	9 (60.00)
Arthralgia	15 (44.12)	11 (32.35)	−	−	6 (40.00)
Nausea and/or vomiting	25 (73.53)	30 (88.24)	+	−	10 (66.67)
Anorexia	33 (97.06)	34 (100.00)	+	−	13 (86.67)
Enlarged lymph nodes	15 (44.12)	0 (0.00)**	−	−	0 (0.00)
Sore throat	9 (33.33)	22 (64.71)*	−	−	7 (46.67)
Oral pharynx red	20 (58.82)	21 (61.76)	−	−	7 (46.67)
Cough	18 (52.94)	8 (23.53)*	−	−	7 (46.67)
Sputum	16 (47.06)	6 (17.65)**	−	−	5 (33.33)
Abnormal breath sound	14 (41.18)	8 (23.53)	−	+	1 (6.67)
Palpitation	7 (20.59)	5 (14.71)	−	−	1 (6.67)
Chest distress	11 (32.35)	10 (29.41)	−	+	1 (6.67)
Abdominal pain	16 (47.06)	14 (41.18)	−	+	3 (20.00)
Epigastric pain	13 (38.24)	11 (32.35)	−	+	3 (20.00)
Diarrhea	13 (38.24)	2 (5.88)**	−	−	3 (18.8)
Melena	4 (11.76)	2 (5.88)	−	−	0 (0)
Renal angle tenderness	10 (29.41)	27 (79.41)**	−	+	4 (26.67)
Oliguria	9 (26.47)	26 (76.47)**	−	−	8 (53.33)
Mental symptom	3 (8.82)	0 (0.00)	−	−	0 (0)
Rashes#	14 (41.18)	22 (64.71)	−	−	5 (33.33)
Petechiae	9 (26.47)	23 (67.65)**	−	+	6 (40.00)
Bleeding	4 (11.76)	3 (8.82)	−	−	1 (6.67)

Abbreviations: HF, hemorrhagic fever; HYSHF, Huaiyangshan hemorrhagic fever; HFRS, hemorrhagic fever with renal syndrome.

# Skin rash refers to erythematous maculopapular lesions that developed on the trunk and/or limbs.

Significant differences observed between HYSHF and HFRS cases (*p<0.05, ** *p*<0.01).

**Figure 2:**
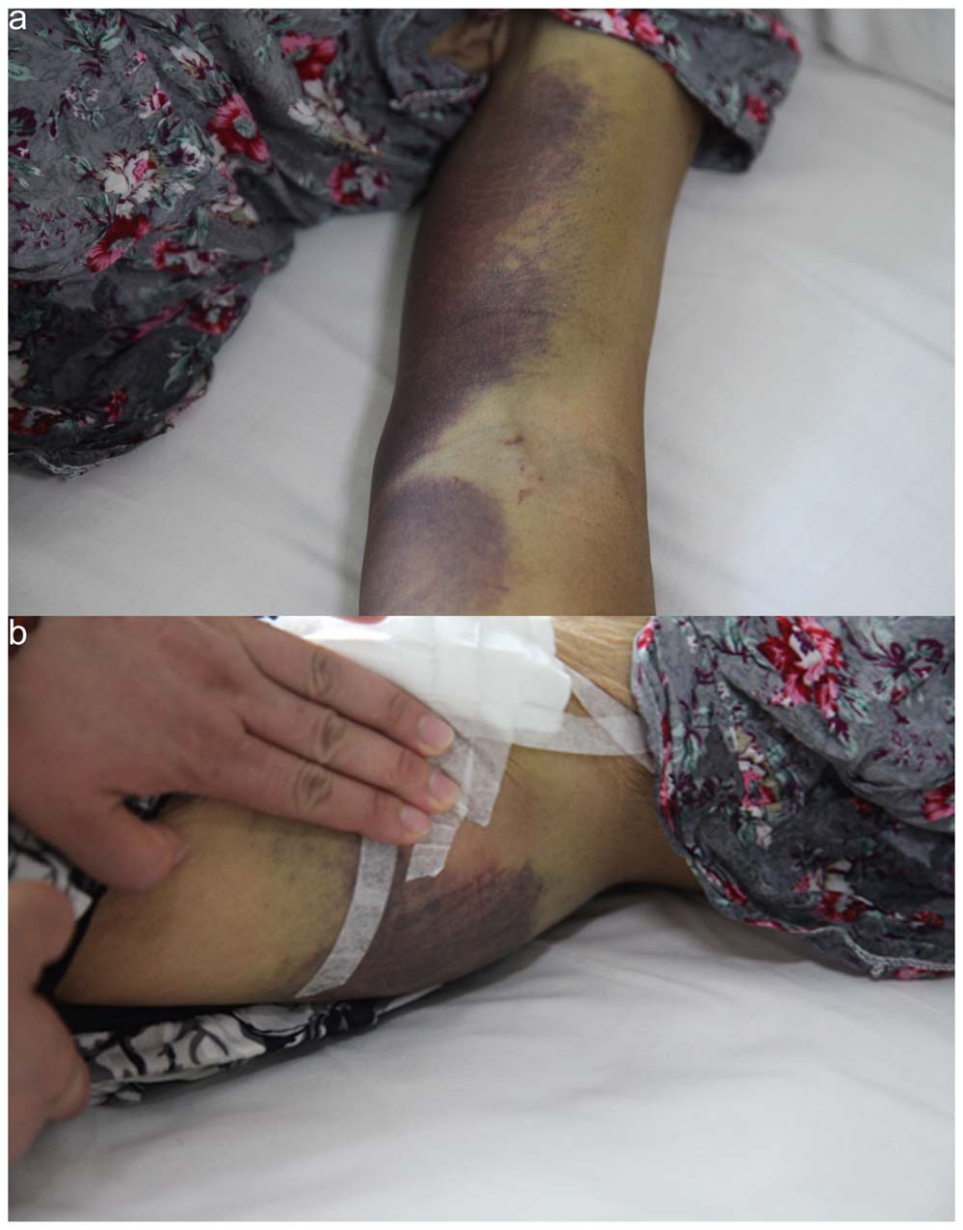
Bleeding exhibited in arm (a) and buttock (b) of a patient infected with HYSV in Dandong.

Two thirds of the HFRS patients had hemorrhagic manifestations. Although the number of HYSHF patients with hemorrhage manifestations was relatively low (Table 4), extensive purpura in arm (Figure 2a) and buttock (Figure 2b) or gastrointestinal tract bleeding were only observed in HYSHF patients. More HYSHF patients had cough, sputum, diarrhea, and enlarged lymph nodes (p<0.05 or p<0.001), while more HFRS patients had headache, sore throat, renal angle tenderness, and oliguria (p<0.05 or p<0.001). In addition, nervous system syndromes occurred in HYSHF patients, but not in HFRS patients.

During the early stage (2–3 days) of onset, most patients began to show significant changes in the blood cell counts (Table 5). Notably, white blood cells (WBC) including neutrophil, lymphocytes, monocytes, and eosinophils decreased dramatically in the majority of HYSHF patients. However, WBC including monocytes increased in most of HFRS patients, while WBC and lymphocytes decreased in nearly half of the unknown infections. Most HYSHF patients displayed a greater elevation of CK levels. High levels of ALT, AST, LDH, and HBDH were observed in most of the HYSHF, HFRS, and unknown infections, but a higher proportion of HYSHF patients showed an elevated ALT, TBA and ADA than HFRS patients (Table 5). Thrombocytopenia occurred in most of HYSV, HV, and unknown infections. Prolonged TT was also observed in most of HYSHF patients and more than half of the HFRS patients. Consistent with the renal dysfunction, the majority of HFRS patients presented with an elevation of blood urea nitrogen (BUN) and creatinine.

**Table 5:** Laboratory parameters of the patients with HF.

Laboratory test	Normal value (NV)	HYSHF		HFRS		Undetermined infections	
		<NV	>NV	<NV	>NV	<NV	>NV
WBC	4–10×10^9^/L	25/32(78.13)**	0/32(0.00)	2/34(5.88)	26/34(76.47)##	7/15(46.67)	4/15(26.67)
NEUT	2–8×10^9^/L	24/32(75.00)**	0/32(0.00)	7/30(23.33)	9/30(30.00)##	5/15(33.33)	3/15(20.00)
LYMPH	1–5×10^9^/L	21/32(65.63)**	0/32(0.00)	9/30(30.00)	8/30(26.67)##	7/15(46.67)	1/15(6.67)
Mono	0.2–0.8×10^9^/L	20/32(62.50)**	4/32(12.50)	8/30(26.67)	21/30(70.00)##	3/15(20.00)	4/15(26.67)
Eos	0.02–0.5×10^9^/L	31/32(96.88)**	0/32(0.00)	6/34(17.65)	1/34(2.94)	8/15(53.33)	1/15(6.67)
RBC	4–5.5×10^12^/L	5/32(15.63)	3/32(9.38)	8/34(23.53)	5/34(14.71)	4/15(26.67)	2/15(13.33)
HGB	120–160 g/L	4/32(12.50)	3/32(9.38)	6/34(17.65)	10/34(29.41)#	4/15(26.67)	3/15(20.00)
PLT	100–300×10^9^/L	31/32(96.88)	1/32(3.13)	33/34(97.06)	0/34(0.00)	13/15(86.67)	0/15(0.00)
LDH	80–285 U/L	0/29(0.00)	27/29(93.10)	0/34(0.00)	32/34(94.12)	0/13(0.00)	9/13(69.23)
CK	38–174 U/L	1/29(3.45)	23/29(79.31)##	6/34(17.65)	10/34(29.41)	1/13(7.69)	4/13(30.77)
CKMB	0–25 U/L	0/29(0.00)	6/29(20.69)	0/34(0.00)	3/34(8.82)	0/13(0.00)	1/13(7.69)
HBDH	74–182 U/L	0/29(0.00)	26/29(89.66)	0/34(0.00)	32/34(94.12)	2/13(15.38)	9/13(69.23)
Na	136–146 mmol/L	22/31(70.97)	0/31(0.00)	29/34(85.29)	0/34(0.00)	7/15(46.67)	0/15(0.00)
Cl	96–108 mmol/L	12/31(38.71)	1/31(3.23)	19/34(55.88)	1/34(2.94)	6/14(42.86)	0/14(0.00)
Ca	2.2–2.55 mmol/L	22/28(78.57)	1/28(3.57)	29/34(85.29)	0/34(0.00)	7/12(58.33)	0/12(0.00)
BUN	1.7–8.3 mmol/L	1/31(3.23)	6/31(19.35)	0/34(0.00)	29/34(85.29)##	0/14(0.00)	3/14(21.43)
CREA	59–104 umol/L	4/31(12.90)	7/31(22.58)	0/34(0.00)	29/34(85.29)##	0/14(0.00)	4/14(28.57)
URCA	202–416 mmol/L	2/27(7.41)	5/27(18.52)	2/30(6.67)	18/30(60.00)##	3/12(25.00)	3/12(25.00)
PT	9–15 s	0/26(0.00)	6/26(23.08)	0/33(0.00)	6/33(18.18)	0/12(0.00)	1/12(8.33)
TT	12–18 s	0/26(0.00)	21/26(80.77)	1/32(3.13)	21/32(65.63)	0/12(0.00)	3/12(25.00)
ALT	0–40 U/L	0/30(0.00)	26/30(86.67)##	0/34(0.00)	19/34(55.88)	0/13(0.00)	9/13(69.23)
AST	0–40 U/L	0/29(0.00)	28/29(96.55)	0/34(0.00)	30/34(88.24)	0/13(0.00)	9/13(69.23)
TBIL	0–18.8 umol/L	0/30(0.00)	6/30(20.00)	0/34(0.00)	3/34(8.82)	0/13(0.00)	4/13(30.77)
DBIL	0–6.8 umol/L	0/28(0.00)	4/28(14.29)	0/33(0.00)	3/33(9.09)	0/13(0.00)	3/13(23.08)
ALB	35–53 g/L	22/30(73.33)*	0/30(0.00)	32/34(94.12)	0/34(0.00)	6/13(46.15)	0/13(0.00)
GLO	27–32 g/L	22/30(73.33)	3/30(10.00)	28/33(84.85)	0/33(0.00)	9/13(69.23)	0/13(0.00)
GGT	7–49 U/L	0/30(0.00)	17/30(56.67)	0/34(0.00)	17/34(50.00)	0/13(0.00)	5/13(38.46)
ALP	42–141 U/L	0/30(0.00)	7/30(23.33)#	2/34(5.88)	1/34(2.94)	0/13(0.00)	2/13(15.38)
TBA	0–10 umol/L	0/30(0.00)	19/30(63.33)#	5/34(14.71)	13/34(38.24)	1/13(7.69)	5/13(38.46)
ADA	4–18 U/L	0/23(0.00)	22/23(95.65)##	1/34(2.94)	14/32(43.75)	0/12(0.00)	6/12(50.00)

Abbreviations: HF, hemorrhagic fever; HYSHF, Huaiyangshan hemorrhagic fever; HFRS, hemorrhagic fever with renal syndrome; WBC, white blood cell; NEUT, neutrophil; LYMPH, lymphocyte; Mono, monocyte; Eos, eosinophil; RBC, red blood cell; HGB, hemoglobin; PLT, platelet; LDH, lactate dehydrogenase; CK, creations kinase; CK-MB, creative kinase isoenzyme MB; HBDH, hydroxyhutyrate dehydrogenase; Na, sodium; Cl, chlorine; Ca, calcium; BUN, blood urea nitrogen; CREA, creatinine; URCA, uric acid; PT, prothrombin time; TT, thrombin time; ALT, alanine aminotransferase; AST, aspartate aminotransferase; TBIL, total bilirubin; DBIL, direct bilirubin; ALB, albumin; GLO, globulin; GGT, gamma-glutamyl transferase; ALP, alkaline phosphatase; TBA, total bile acids; ADA, adenosine deaminase.

Significant differences were observed between HYSHF and HFRS cases (*p<0.05, **p<0.01; #p<0.05 ##p<0.01).

### Changes in temperature, WBC, and biochemical parameters

**Figure 3:**
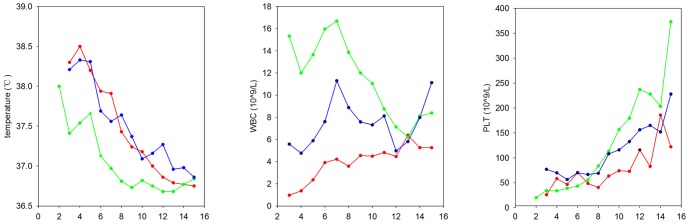
Dynamic profiles of temperature, white blood cell (WBC), and platelet (PLT) counts in patients with HYSHF (red line), HFRS (green line), or undetermined infections (blue line). More details are given in Table S3.

To better understand the clinical course of HF, we further compared the changes in temperature, blood cells, and biochemical parameters during progression (from day 1 to day 15 during hospitalization) (Figure 3). During the observation period both relatively high temperatures (0.25–0.96°C) and a longer duration of high temperature were observed in HYSHF compared to HFRS patients (Figure 3, Table S3). WBC levels in the HYSHF patients decreased dramatically during days 3 to 5 after the onset of the disease (Figure 3), while WBC levels in the HFRS patients increased significantly above the normal level during days 3 to 10. Notably, WBC levels were normal in the patients with undetermined infection(s) during this period. For HFRS and undetermined infection(s), platelet counts were lower than normal levels during days 2 to 9, and even up to day 11 for the HYSHF patients.

**Figure 4:**
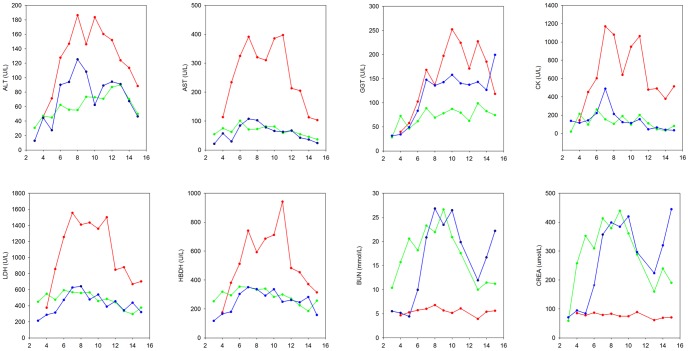
Changes in alanine aminotransferase (ALT), aspartate aminotransferase (AST), gamma-glutamyl transferase (GGT), creatine kinase (CK), lactate dehydrogenase (LDH), hydroxyhutyrate dehydrogenase (HBDH), blood urea nitrogen (BUN), and creatinine (CREA) levels in patients with HYSHF, HFRS, and undetermined infections. The color coding is the same as in Figure 3. More details are given in Table S4.

We also analyzed the changes in biochemical parameters for HYSHF, HFRS, and undetermined infections (Figure 4). Elevations of ALT, AST, and GGT, which are the main index of liver damage, were observed in HYSHF, HFRS, and infections of undetermined cause. Notably, the elevation (especially AST) was greater in HYSHF than in HFRS patients during day 5 to day 14, suggesting that liver damage might be more severe in HYSHF than in HFRS. Although high levels of LDH, CK, and HBDH were found in HFRS patients and those with infections of undetermined etiology, a dramatic increase was observed in HYSHF patients during disease progression (Figure 4, Table S4). Consistent with the renal damage of HFRS patients, the levels of BUN and CREA were significantly higher than normal during days 3–15 in HFRS patients (Figure 4). The infections of unknown etiology also displayed a higher level of BUN and CREA during the days 6 to 11 after onset.

### Treatment and outcomes

Several strategies were implemented for the treatment of patients with suspected HFs. First, because most patients were in a critical condition due to the loss of blood and body fluids, all patients were placed on supportive therapy (including providing electrolytes and glucose) to maintain the correct balance of body fluids and electrolytes, and to support bodily functions. Second, as ribavirin is currently recommended for prophylaxis and treatment of viral diseases such as HFRS, CCHF, Lassa fever [2], all HFRS patients diagnosed by clinicians in Dandong Infectious Disease Hospital (a total of 23, or 67.6% of total laboratory confirmed 34 cases) were treated with an intravenous administration of 0.6 g ribavirin once a day. Six patients with suspected HYSHF and nine with suspected other viral infections also received the drug. Third, antibiotics such as doxycycline were used to treat those patients who were initially diagnosed as rickettsial diseases and to prevent secondary bacterial infection(s) because some patients developed neutropenia and lymphopenia. Finally, broad-spectrum antibiotics, granulocyte colony- stimulating factor (G-CSF) and other supportive measures were applied when needed. Despite all these measures, five patients died; two from HYSHF, two from HFRS, and one from an as yet undetermined infection. At the point of admission all these five patients were clinically diagnosed with undetermined infections. Notably, of these five deaths, two HFRS and one HYSHF patients did not receive an intravenous administration of ribavirin.

## Discussion

Hemorraghic fevers are a diverse group of serious human diseases caused by RNA viruses and a variety of *Rickettsiales* bacteria [2]–[4]. In this study we observed HYSV, HV, *R. typhi*, and *O. tsutsugamushi* infections in the city of Dandong, Liaoning Province, northern China. Interestingly, the etiologic agent could not be identified in 15 patients with HF. Hence, these data reveal the complexity of pathogens causing HFs and also suggest the possible presence of unknown human pathogens in this geographic region. It is therefore clear that additional studies are needed to establish appropriate diagnostic tests.

Currently, there are no commercial kits for the diagnosis of HFs, and specific diagnostics are only available in specialized laboratories [2]. Almost all hospitals in China lack the specific reagents for the diagnosis of HFs with the exception of HFRS, and which clearly contributes to the high rate of misdiagnoses observed here. Furthermore, the absence of distinctive clinical signs and symptoms at the early stage of disease enhances misdiagnosis, even for HFRS [41]. In addition, milder or asymptomatic cases may exist and which would be missed by sentinel surveillance. Thus, the true numbers of HYSHF cases might be several times higher than those reported. Hence, our data highlight the urgent need for diagnostic reagents, while a better understanding of the clinical features of each HF disease is also likely to improve their diagnosis.

We compared the epidemiologic characteristics of emerging HYSHF with HFRS in the same population using matching enrollment, laboratory diagnostics and the follow-up. Consistent with early reports [15], all HYSHF cases occurred from June to September, while all HFRS cases caused by HTNV were recorded in the winter, similarly to previous reports [9], [42]. These data suggest that human behavior in the summer likely increases tick exposure risk [33], while cold conditions during winter increase rodent infestations of houses [42]. Hence, disease seasonality may provide some useful pointers for diagnosis. Because of favorable ecological conditions and low socioeconomic status in rural areas, farmers have frequently been the major victims of zoonoses including HFRS, scrub typhus, and leptospirosis [5], [9], [10], [14], [43]. Thus, our data strongly reinforces the need for vigilance in preventing the spillover of HV, HYSV from animals in the rural areas of China.

Like other hantaviruses (e.g. PUUV) [44], [45], HTNV exhibits high genetic diversity with at least nine lineages present [34]. Furthermore, it displays a marked geographic clustering of genetic variants, especially in mountainous regions [34]. In this study, the S and M segment sequences recovered from 24 HFRS patients were divided into two groups, and shared a close phylogenetic relationship with those viruses recovered from HFRS patients and *Apodemus* mice from Far East Asia [1], [34]–[40], indicating that human infection is associated with the spillover of HTNV. Although the HYSV sequences recovered in this study were very closely related to each other, they could be divided into three lineages in the S and L trees (Figure 1ab) [33], indicative of a relatively high genetic diversity of HYSV in Dandong. Interestingly, these sequences also shared a close relationship with those recovered from ticks and humans sampled from Anhui, Hubei, Jiangsu, and Shandong provinces, with a lack of geographic structure that may reflect the recent emergence and spread of this virus.

The severity and clinical presentation of viral HF diseases were related to the etiologic agents involved [3]. Among the known etiologic agents of HFRS, HTNV and Dobrava-Belgrade virus (DOBV) can cause a severe form of HFRS, with up to a 15% fatality rate [5], [41], [43], [45], while SEOV usually causes a milder form of HFRS with a mortality rate of approximately 1% [5], [41]. In contrast, PUUV only causes a mild disease referred to as nephropathia epidemica (NE) with a mortality rate of <0.3% in Europe [43], [46]. Serological and PCR tests indicated that of the viruses causing HFRS in Dandong almost all belonged to HTNV with the exception of one patient infected by SEOV, and the clinical manifestations were similar to those of HFRS caused by HTNV as described previously [1], with two patients dying. Phylogenetic analyses of viral sequences showed that the HTNV variants described in this study were closely related to those (CGHu1, Hojo, Lee, Maaji-2) causing severe HFRS in China and other part of Far East Asia [1], [34]. Clearly, more effort is needed to prevent severe HFRS caused by HTNV in this region of China.

Similar to HFRS and other viral HF diseases [1], [3], the onset of HYSHF is characterized by malaise, anorexia, chills, headache, myalgias, and fever [15], [16]. A few days after onset patients develop constitutional, gastrointestinal, cardiovascular, and neurological signs and symptoms. We compared clinical signs and biochemical parameters, as well as their dynamics, in HYSHF and HFRS patients. Clearly, temperatures in the HYSHF patients were relatively higher and continued to be high for longer than in the HFRS patients. More serious bleeding was also observed in the HYSHF patients, but not in the HFRS patients. In addition, the HYSHF patients presented with more serious hepatic injury than HFRS, as manifest in a dramatic elevation of ALT. Experiments using animal models revealed larger necrotic areas and greater mononuclear cell infiltration in the liver of HYSV-infected mice rather than HTNV-infected mice [47]. In sum, these data support the definition, “Huaiyangshan hemorrhage fever”, for the novel disease with high fever and hemorrhagic manifestations that was first reported in the Huaiyangshan region of China [15], and which is clinically comparable to HFRS.

Currently, the treatment of viral HF, including HYSHF, involves the use of specific antivirals and general supportive measures [2], [19], [48]. In this study, all suspected patients (38 cases) with viral HF received supportive therapy when needed and were treated with ribavirin intravenously. In addition, antibiotics including doxycycline were used to prevent secondary bacterial infection(s). Despite these measures, two HYSHF and two HFRS patients died. Thus, there is an urgent need to develop effective clinical protocols for patients with HF of viral origin, especially for HFRS and HYSHF in China.

In conclusion, HYSV and HV are prevalent in humans in Dandong. Murine typhus and scrub typhus were also reported. Importantly, the etiologic agents of HFs could not be determined in all cases. These data therefore highlight the complexity of the pathogens that cause human HFs. Moreover, most of the clinical symptoms for HYSHF were comparable to those of HFRS, with their distinctive clinical features not developing until later in disease progression.

## Supporting Information

Figure S1 **Map of Liaoning province showing the location of Dandong City.**
(TIF)

Figure S2 **The seasonal distribution of HYSHF and HFRS cases reported during 2011–2012 in Dandong. The color code is the same as** **Figure 3** **.**
(TIF)

Table S1 **The clinical and laboratory criteria listed by the Ministry of Health, China for HYSHF, HFRS, murine typhus, scrub typhus, and other related diseases.**
(DOCX)

Table S2 **The viral and bacterial sequences recovered in this study and those taken from GenBank.**
(DOC)

Table S3 **Dynamic profile of the body temperature, WBC, and PLT in patients with HYSHF, HFRS, and undetermined infections.**
(DOCX)

Table S4 **Dynamic profile of the ALT, AST, GGT, CK, LDH, HBDH, BUN, and CREA in patients with HYSHF, HFRS, and undetermined infections.**
(DOCX)
