# Supplementary figures and images for: Co-Circulation of Multiple Hemorrhagic Fever Diseases with Distinct Clinical Characteristics in Dandong, China
Source: PLoS One. 2014 Feb 27;9(2):e89896. doi: 10.1371/journal.pone.0089896 (PMC3937409; doi:10.1371/journal.pone.0089896)

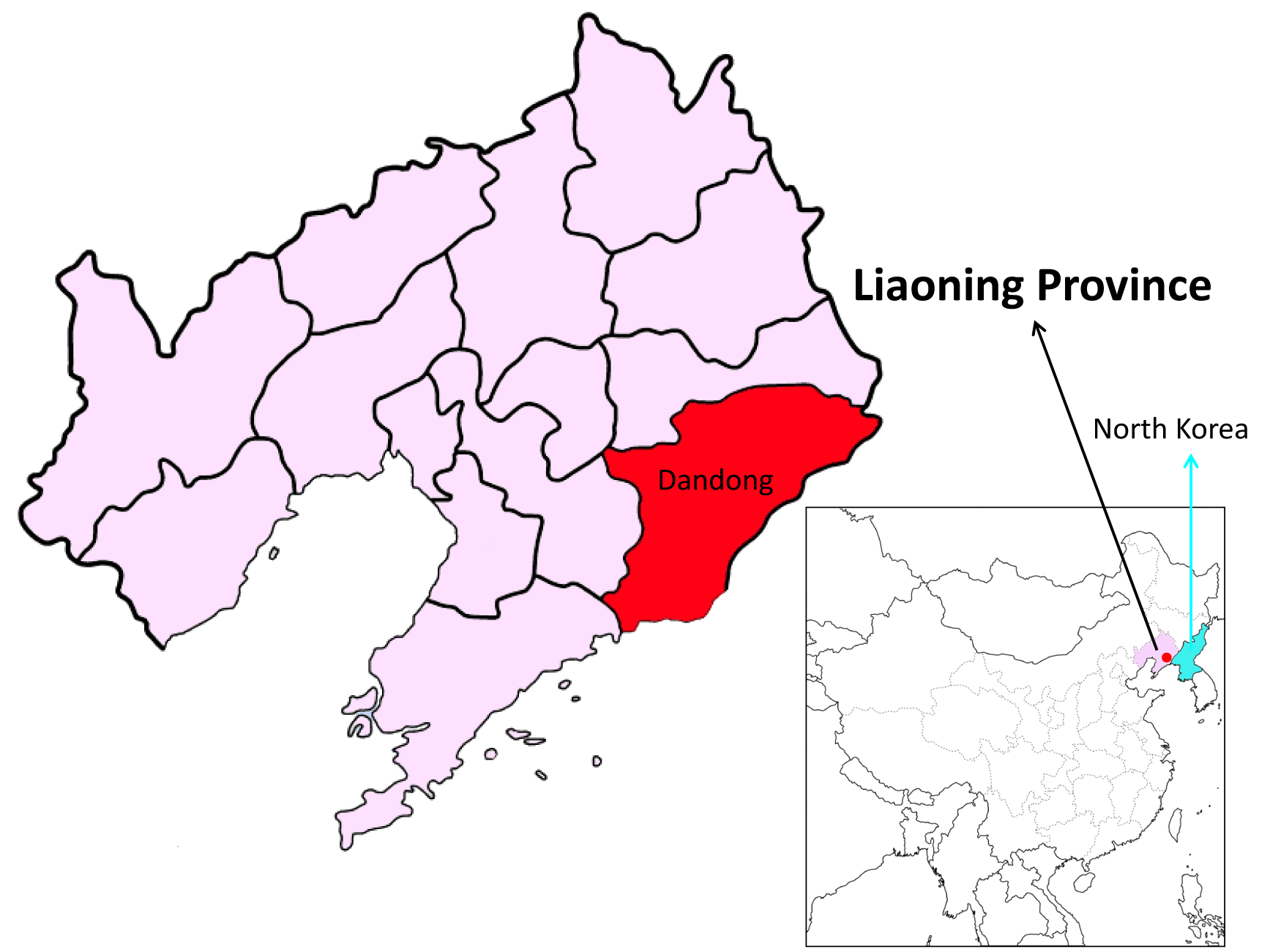

Supplement: Figure S1 — Map of Liaoning province showing the location of Dandong City. (TIF) [file pone.0089896.s001.tif]

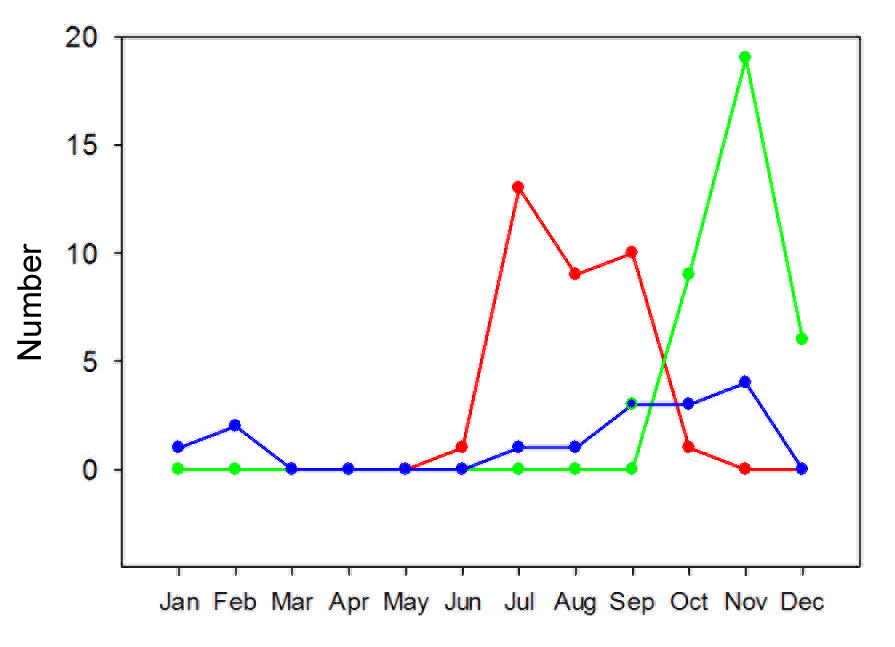

Supplement: Figure S2 — The seasonal distribution of HYSHF and HFRS cases reported during 2011–2012 in Dandong. The color code is the same as Figure 3 . (TIF) [file pone.0089896.s002.tif]
