# Supplementary material for: Co-Circulation of Multiple Hemorrhagic Fever Diseases with Distinct Clinical Characteristics in Dandong, China
Source: PLoS One. 2014 Feb 27;9(2):e89896. doi: 10.1371/journal.pone.0089896 (PMC3937409; doi:10.1371/journal.pone.0089896)
Supplement: Table S1 — The clinical and laboratory criteria listed by the Ministry of Health, China for HYSHF, HFRS, murine typhus, scrub typhus, and other related diseases. (DOCX) [file pone.0089896.s003.docx]

| Clinical Features | HYSHF | HFRS | | Scrub typhus | | | Murine typhus |
| --- | --- | --- | --- | --- | --- | --- | --- |
| Fever | Yes | | Yes | | Yes | Yes | |
| Dizziness | Yes | | Yes | | Yes | - | |
| Myalgia | Yes | | Yes | | Yes | - | |
| Nausea | Yes | | Yes | | Yes | - | |
| Vomiting | Yes | | Yes | | Yes | - | |
| Anorexia | Yes | | Yes | | Yes | - | |
| Dizziness | Yes | | Yes | | Yes | - | |
| Mental symptom | Yes | | - | | Yes | Yes | |
| Rash | Erythema, maculopapule | | Congestion, flushing in face, neck and chest | | Eschar, erythema maculopapule | Erythema maculopapule | |
| Petechiae | Yes (mucosa, skin) | | Yes | | Yes | Yes | |
| Bleeding | In gastrointestinal and respiratory tracts | | Yes | | - | - | |
| Enlarged lymph nodes | Often in neck and groin | | Seldom | | Near eschar areas | Seldom | |
| Biochemical parameters | | | | | | | |
| WBC(4-10×10^9^/L) | <4 | | >10 | | 4-10 | - | |
| PLT(100-300×10^9^/L) | <100 | | <100 | | <100 | - | |
| Routine Urine test | Abnormal | | Abnormal | | Abnormal | - | |
| ALT(0-40 U/L) | >40 | | - | | >40 | - | |
| AST(0-40 U/L) | >40 | | - | | >40 | - | |
| CK(38-174 U/L) | >174 | | - | | - | - | |
| CK-MB(0-25 U/L) | >25 | | - | | - | - | |
| LDH(80-285 U/L) | >285 | | - | | - | - | |
| Na(136-146 mmol/L) | <136 | | - | | - | - | |
| BUN(1.7-8.3mmol/L) | >8.3 | | >8.3 | | - | - | |
| CREA(59-104umol/L) | >104 | | >104 | | - | - | |

Table S1. The clinical and laboratory criteria listed by the Ministry of Health, China for HYSHF, HFRS, murine typhus, scrub typhus, and other related diseases.

Abbreviations: WBC, white blood cell; PLT, platelet; ALT, alanine aminotransferase; AST, aspartate aminotransferase; CK, creations kinase; CK-MB, creative kinase isoenzyme MB; LDH, lactate dehydrogenase; Na, sodium; BUN, blood urea nitrogen; CREA, creatinine.

-, not defined.
