# Supplementary material for: Co-Circulation of Multiple Hemorrhagic Fever Diseases with Distinct Clinical Characteristics in Dandong, China
Source: PLoS One. 2014 Feb 27;9(2):e89896. doi: 10.1371/journal.pone.0089896 (PMC3937409; doi:10.1371/journal.pone.0089896)
Supplement: Table S2 — The viral and bacterial sequences recovered in this study and those taken from GenBank. (DOC) [file pone.0089896.s004.doc]

**Table S2.** The viral and bacterial sequences recovered in this study and those taken from GenBank.

| Strain | S segment | M segment | L segment | 16S rRNA |
| --- | --- | --- | --- | --- |
| Rift valley fever virus | DQ380149^#^ | − | DQ375406^#^ | − |
| Toscana virus | NC_006318^#^ | − | NC_006319^#^ | − |
| Uukuniemi virus | M33551^#^ | − | D10759^#^ | − |
| 2010-24 | HQ179752^#^ | − | HQ179718^#^ | − |
| 2010-29 | HQ179753^#^ | − | HQ179719^#^ | − |
| 2010-32 | HQ179754^#^ | − | HQ179720^#^ | − |
| 2010-37 | HQ179757^#^ | − | HQ179723^#^ | − |
| 2010-39 | HQ179747^#^ | − | HQ179715^#^ | − |
| 2010-51 | HQ179761^#^ | − | [HQ179727](http://www.ncbi.nlm.nih.gov/nucleotide/327177459?report=genbank&log$=nucltop&blast_rank=1&RID=GP6T7E7F015)^#^ | − |
| Shandong 4 | HQ802204^#^ | − | HM802202^#^ | − |
| Shandong 24 | HM802200^#^ | − | HM802205^#^ | − |
| Hubei 29 | HM745932^#^ | − | HM745930^#^ | − |
| Jaingsu 3 | HQ141603^#^ | − | HQ141601^#^ | − |
| Jiansu 4 | HQ141606^#^ | − | HQ141604^#^ | − |
| Anhui 12 | HQ141591^#^ | − | HQ116417^#^ | − |
| Anhui 15 | HQ141594^#^ | − | HQ141592^#^ | − |
| Liaoning 3 | HQ141612^#^ | − | HQ141610^#^ | − |
| Heartland virus 1 | JX005842^#^ | − | JX005846^#^ | − |
| Heartland virus2 | JX005843^#^ | − | JX005847^#^ | − |
| DandongHu-1 | KC570422 | − | − | − |
| Dandong Hu-2 | − | − | KC570408 | − |
| DandongHu-6 | KC570423 | − | KC570409 | − |
| DandongHu-7 | KC570424 | − | − | − |
| DandongHu-8 | KC570425 | − | − KC570410 | − |
| DandongHu-9 | KC570426 | − | − | − |
| DandongHu-11 | KC570427 | − | − KC570411 | − |
| DandongHu-12 | KC570428 | − | − | − |
| DandongHu-13 | KC570429 | − | KC570412 | − |
| DandongHu-14 | KC570430 | − | − | − |
| DandongHu-15 | KC570431 | − | KC570413 | − |
| DandongHu-17 | KC570432 | − | KC570414 | − |
| DandongHu-18 | KC570433 | − | − | − |
| DandongHu-19 | KC570434 | − | KC570415 | − |
| DandongHu-20 | KC570435 | − | KC570416 | − |
| DandongHu-54 | KC570436 | − | − | − |
| DandongHu-55 | KC570437 | − | − | − |
| DandongHu-56 | KC570438 | − | KC570417 | − |
| DandongHu-57 | KC570439 | − | KC570418 | − |
| DandongHu-58 | KC570440 | − | − | − |
| DandongHu-60 | KC570441 | − | − | − |
| DandongHu-61 | KC570442 | − | KC570419 | − |
| DandongHu-62 | KC570443 | − | − | − |
| DandongHu-63 | KC570444 | − | − | − |
| DandongHu-64 | KC570445 | − | − | − |
| DandongHu-66 | KC570446 | − | − | − |
| DandongHu-69 | KC570447 | − | − | − |
| DandongHu-70 | KC570448 | − | KC570420 | − |
| DandongHu-72 | KC570449 | − | KC570421 |  |
| DandongHu-75 | KC570450 | − | − | − |
| 76-118 | M14626^#^ | Y00386^#^ | − | − |
| 84FLi | AY017064^#^ | AF345636^#^ | − | − |
| A9 | AF329390^#^ | AF035831^#^ | − | − |
| Bao14 | AB127998^#^ | AB127995^#^ | − | − |
| CFC94-2 | X95077^#^ | − | − | − |
| CGAa1011 | EF990913^#^ | EF990927^#^ | − | − |
| CGHu1 | EU092218^#^ | EU092222^#^ | − | − |
| Chen4 | AB027101^#^ | − | − | − |
| CJAp93 | EF208929^#^ | EF208930^#^ | − | − |
| CUMC-B11 | U37768^#^ | U38177^#^ | − | − |
| E142 | AF288644^#^ |  | − | − |
| Galkino/AA57/2002 | AB620031^#^ | AB620032^#^ | − | − |
| HoJo | − | D00376 | − | − |
| HTN261 | AF252259 | − | − | − |
| HV114 | − | L08753 | − | − |
| KY | GU140098^#^ | GU140097^#^ | − | − |
| Lee | − | D00376 | − | − |
| LR1 | AF288294^#^ | AF288293^#^ |  |  |
| Maaji-2 | AF321095^#^ | − | − | − |
| S85-46 | AF288659^#^ | AF288658^#^ | − | − |
| XAHu09066 | JF421284 ^#^ | − | − | − |
| YaluRiver13 | HQ611981^#^ | − | − | − |
| Z251 | EF595840^#^ | GQ120966^#^ | − | − |
| Z5 | EF103195^#^ | EU074224^#^ | − | − |
| B78 | AB127997^#^ | AB127994^#^ | − | − |
| H5 | AB127996^#^ | AB127993^#^ | − | − |
| H8205 | − | AB030232^#^ | − | − |
| Liu | AF288649^#^ | AF288648^#^ | − | − |
| SC1 | AY675349^#^ | AY675353^#^ | − | − |
| Wencheng-Nc-427 | JF796017^#^ | JF796031^#^ | − | − |
| DandongHu-22 | KC570384^*^ | − | − | − |
| DandongHu-28 | KC570385^*^ | − | − | − |
| DandongHu-32 | KC570386^*^ | − | − | − |
| DandongHu-34 | KC570387^*^ | − | − | − |
| DandongHu-44 | KC570388^*^ | KC570378 | − | − |
| DandongHu-89 | KC570389^*^ | − | − | − |
| DandongHu-91 | KC570390^*^ | KC570383 | − | − |
| DandongHu-27 | KC570391 | − | − | − |
| DandongHu-29 | KC570392 | − | − | − |
| DandongHu-33 | KC570393 | − | − | − |
| DandongHu-36 | KC570394 | − | − | − |
| DandongHu-42 | KC570395 | KC570377 | − | − |
| DandongHu-47 | KC570396 | − | − | − |
| DandongHu-77 | KC570397 | KC570379 | − | − |
| DandongHu-78 | KC570398 | − | − | − |
| DandongHu-82 | KC570399 | KC570380 | − | − |
| DandongHu-83 | KC570400 | KC570381 | − | − |
| DandongHu-84 | KC570401 | − | − | − |
| DandongHu-85 | KC570402 | − | − | − |
| DandongHu-86 | KC570403 | − | − | − |
| DandongHu-88 | KC570404 | KC570382 | − | − |
| DandongHu-90 | KC570405 | − | − | − |
| DandongHu-92 | KC570406 | − | − | − |
| DandongHu-93 | KC570407 | − | − | − |
| DandongHu-81 | − | − | − | KC756843 |
| *O. tsutsugamushi* Boryong | − | − | − | NC_009488 |
| *O. tsutsugamushi* Ikeda | − | − | − | NC_010793 |
| *O. tsutsugamushi* 07-280 | − | − | − | HM352765 |
| *O. tsutsugamushi* CB35 | − | − | − | GU068051 |
| *O. tsutsugamushi* CB49 | − | − | − | GU068050 |
| *O. tsutsugamushi* Karp | − | − | − | NR_025860 |
| *O. tsutsugamushi* Kato | − | − | − | DQ372907 |
| *O. tsutsugamushi* TA686 | − | − | − | AF479302 |
| *O. tsutsugamushi* TC586 | − | − | − | AF479303 |
| *O. tsutsugamushi* TA678 | − | − | − | AF479301 |
| *O. tsutsugamushi* TA763 | − | − | − | AF479299 |
| *O. tsutsugamushi* Gilliam | − | − | − | L36222 |
| *O. tsutsugamushi* Kawasaki | − | − | − | D38625 |
| *O. tsutsugamushi* Kuroki | − | − | − | D38626 |
| *O. tsutsugamushi* Shimokoshi | − | − | − | D38627 |
| *O. tsutsugamushi* Litchfield | − | − | − | AF062074 |
| *O. tsutsugamushi* TH1817 | − | − | − | AF479300 |
| *R. typhi* | − | − | − | U12463 |
| *R. raoultii* | − | − | − | EU036982 |
| *R.endosymbiont of* Lutzomyia.apache | − | − | − | EU200324 |

*Complete genome obtained in this study.

#GenBank accession numbers.
