# Supplementary material for: Co-Circulation of Multiple Hemorrhagic Fever Diseases with Distinct Clinical Characteristics in Dandong, China
Source: PLoS One. 2014 Feb 27;9(2):e89896. doi: 10.1371/journal.pone.0089896 (PMC3937409; doi:10.1371/journal.pone.0089896)
Supplement: Table S3 — Dynamic profile of the body temperature, WBC, and PLT in patients with HYSHF, HFRS, and undetermined infections. (DOCX) [file pone.0089896.s005.docx]

**Table S3-1** Dynamic profile of the body temperature in patients with HYSHF, HFRS, and undetermined infection.

| Course  (day) | HYSHF | HFRS | Undetermined infection |
| --- | --- | --- | --- |
|  | Mean ± SD | Mean ± SD | Mean ± SD |
| 1 | - | - | - |
| 2 | - | 38.00±0.00 | - |
| 3 | 38.30±1.02 | 37.41±0.84 | 38.21±0.47 |
| 4 | 38.50±0.53 | 37.54±0.75 | 38.33±1.11 |
| 5 | 38.20±0.85 | 37.66±1.08 | 38.31±1.14 |
| 6 | 37.94±0.75 | 37.13±0.70 | 37.69±1.23 |
| 7 | 37.91±0.79 | 36.97±0.67 | 37.56±1.37 |
| 8 | 37.43±0.87 | 36.81±0.41 | 37.64±1.46 |
| 9 | 37.24±0.74 | 36.73±0.34 | 37.37±1.13 |
| 10 | 37.18±0.80 | 36.82±0.61 | 37.09±0.57 |
| 11 | 37.00±0.57 | 36.75±0.72 | 37.16±0.77 |
| 12 | 36.86±0.47 | 36.68±0.41 | 37.27±1.12 |
| 13 | 36.79±0.40 | 36.68±0.40 | 36.96±0.93 |
| 14 | 36.77±0.32 | 36.77±0.47 | 36.98±0.69 |
| 15 | 36.75±0.26 | 36.84±0.74 | 36.86±0.52 |

Abbreviations: HYSHF: Huaiyangshan hemorrhagic fever, HFRS: hemorrhagic fever with renal syndrome.

-: not available.

**Table S3-2** Dynamic profile of the WBC in patients with HYSHF, HFRS, and undetermined infection.

| Course  (day) | HYSHF | HFRS | Undetermined infection |
| --- | --- | --- | --- |
|  | Mean ± SD | Mean ± SD | Mean ± SD |
| 1 | - | - | - |
| 2 | - | - | - |
| 3 | 0.97±0.00 | 15.33±5.94 | 5.58±4.73 |
| 4 | 1.36±0.75 | 12.00±5.09 | 4.76±5.00 |
| 5 | 2.35±1.89 | 13.66±5.83 | 5.89±7.64 |
| 6 | 3.91±3.46 | 15.97±13.19 | 7.61±7.46 |
| 7 | 4.22±3.38 | 16.69±11.55 | 11.30±9.02 |
| 8 | 3.59±2.36 | 13.87±11.19 | 8.88±7.50 |
| 9 | 4.55±3.27 | 12.00±9.95 | 7.58±4.83 |
| 10 | 4.49±3.65 | 11.05±6.81 | 7.32±4.74 |
| 11 | 4.82±2.23 | 8.75±4.18 | 8.12±3.97 |
| 12 | 4.46±2.15 | 7.12±2.91 | 4.97±3.52 |
| 13 | 6.38±5.38 | 6.23±2.44 | 5.81±4.33 |
| 14 | 5.26±1.52 | 8.11±4.39 | 7.98±2.29 |
| 15 | 5.26±2.29 | 8.39±2.78 | 11.13±0.00 |

Abbreviations: HYSHF: Huaiyangshan hemorrhagic fever, HFRS: hemorrhagic fever with renal syndrome.

-: not available.

**Table S3-3** Dynamic profile of the PLT in patients with HYSHF, HFRS, and undetermined infection.

| Course  (day) | HYSHF | HFRS | Undetermined infection |
| --- | --- | --- | --- |
|  | Mean ± SD | Mean ± SD | Mean ± SD |
| 1 | - | - | - |
| 2 | - | 20.00±0.00 | - |
| 3 | 26.00±0.00 | 34.20±15.59 | 77.00±11.61 |
| 4 | 58.20±36.47 | 33.74±20.33 | 69.83±43.82 |
| 5 | 46.55±12.06 | 39.00±22.38 | 56.43±45.56 |
| 6 | 70.46±93.82 | 43.26±21.74 | 70.04±63.20 |
| 7 | 48.67±33.18 | 55.69±44.78 | 66.63±57.65 |
| 8 | 40.55±14.49 | 83.68±57.81 | 69.14±80.93 |
| 9 | 63.45±77.99 | 113.28±80.50 | 108.05±109.30 |
| 10 | 74.09±88.54 | 156.77±94.24 | 115.89±117.17 |
| 11 | 72.94±113.79 | 179.58±97.27 | 132.67±120.00 |
| 12 | 115.88±139.05 | 237.22±140.79 | 156.30±156.52 |
| 13 | 82.86±58.42 | 227.96±129.39 | 165.05±110.08 |
| 14 | 185.83±142.53 | 203.32±105.85 | 152.00±80.61 |
| 15 | 122.11±77.48 | 373.18±127.95 | 228.00±0.00 |

Abbreviations: HYSHF: Huaiyangshan hemorrhagic fever, HFRS: hemorrhagic fever with renal syndrome.

-: not available.
