# Supplementary material for: Co-Circulation of Multiple Hemorrhagic Fever Diseases with Distinct Clinical Characteristics in Dandong, China
Source: PLoS One. 2014 Feb 27;9(2):e89896. doi: 10.1371/journal.pone.0089896 (PMC3937409; doi:10.1371/journal.pone.0089896)
Supplement: Table S4 — Dynamic profile of the ALT, AST, GGT, CK, LDH, HBDH, BUN, and CREA in patients with HYSHF, HFRS, and undetermined infections. (DOCX) [file pone.0089896.s006.docx]

Table S4-1 Dynamic profile of the ALT in patients with HYSHF, HFRS, and undetermined infection.

| Course  (day) | HYSHF | HFRS | Undetermined infection |
| --- | --- | --- | --- |
|  | Mean ± SD | Mean ± SD | Mean ± SD |
| 1 | - | - | - |
| 2 | - | - | - |
| 3 | - | 30.60±0.00 | 12.80±0.00 |
| 4 | 46.17±40.96 | 47.19±33.11 | 44.42±20.77 |
| 5 | 71.37±49.09 | 44.87±36.51 | 27.33±21.04 |
| 6 | 127.73±63.05 | 62.41±52.13 | 90.15±45.42 |
| 7 | 146.94±111.72 | 55.61±47.68 | 94.10±77.52 |
| 8 | 186.42±208.74 | 55.17±37.28 | 125.35±167.10 |
| 9 | 146.21±117.33 | 73.49±44.25 | 108.30±119.89 |
| 10 | 183.63±123.43 | 72.91±67.23 | 62.32±35.40 |
| 11 | 160.39±80.78 | 70.86±54.30 | 89.10±97.61 |
| 12 | 152.08±94.35 | 87.03±48.57 | 94.43±15.64 |
| 13 | 124.00±68.54 | 89.81±54.09 | 91.16±32.21 |
| 14 | 113.56±63.77 | 70.22±60.73 | 67.60±65.50 |
| 15 | 88.43±53.47 | 49.93±37.25 | 46.40±3.82 |

Abbreviations: HYSHF: Huaiyangshan hemorrhagic fever, HFRS: hemorrhagic fever with renal syndrome.

-: not available.

Table S4-2 Dynamic profile of the AST in patients with HYSHF, HFRS, and undetermined infection.

| Course  (day) | HYSHF | HFRS | Undetermined infection |
| --- | --- | --- | --- |
|  | Mean ± SD | Mean ± SD | Mean ± SD |
| 1 | - | - | - |
| 2 | - | - | - |
| 3 | - | 54.30±0.00 | 21.30±0.00 |
| 4 | 113.60±130.51 | 75.08±56.92 | 57.23±29.22 |
| 5 | 234.10±243.38 | 62.53±47.86 | 29.43±17.91 |
| 6 | 325.12±182.57 | 101.16±80.82 | 84.48±47.63 |
| 7 | 391.35±375.68 | 70.99±55.58 | 107.70±65.22 |
| 8 | 320.55±228.71 | 72.31±65.70 | 102.63±103.67 |
| 9 | 310.35±267.09 | 81.57±62.39 | 78.12±43.61 |
| 10 | 385.79±407.32 | 81.08±77.01 | 65.84±39.62 |
| 11 | 397.26±322.10 | 58.89±43.25 | 62.79±42.69 |
| 12 | 213.25±218.65 | 66.41±22.63 | 67.20±2.52 |
| 13 | 204.95±188.91 | 54.28±26.88 | 42.26±8.14 |
| 14 | 113.02±83.08 | 45.57±35.22 | 36.07±20.86 |
| 15 | 103.25±75.47 | 36.65±19.41 | 24.05±18.46 |

Abbreviations: HYSHF: Huaiyangshan hemorrhagic fever, HFRS: hemorrhagic fever with renal syndrome.

-: not available.

Table S4-3 Dynamic profile of the GGT in patients with HYSHF, HFRS, and undetermined infection.

| Course  (day) | HYSHF | HFRS | Undetermined infection |
| --- | --- | --- | --- |
|  | Mean ± SD | Mean ± SD | Mean ± SD |
| 1 | - | - | - |
| 2 | - | - | - |
| 3 | - | 28.20±0.00 | 31.50±0.00 |
| 4 | 39.53±25.28 | 72.41±56.84 | 34.27±16.01 |
| 5 | 57.87±52.14 | 46.70±23.00 | 49.20±29.48 |
| 6 | 102.48±167.66 | 61.66±65.77 | 83.37±104.31 |
| 7 | 168.22±219.28 | 88.54±85.43 | 147.67±147.90 |
| 8 | 137.19±195.28 | 69.23±61.73 | 135.80±125.95 |
| 9 | 197.26±324.92 | 78.17±57.28 | 142.73±96.01 |
| 10 | 252.33±277.74 | 87.17±54.78 | 157.92±73.57 |
| 11 | 224.41±232.73 | 79.51±50.94 | 140.24±82.02 |
| 12 | 170.85±190.47 | 62.45±34.56 | 137.37±37.60 |
| 13 | 227.42±191.89 | 98.85±62.93 | 143.20±75.43 |
| 14 | 185.23±148.88 | 82.54±52.51 | 126.67±98.53 |
| 15 | 118.41±89.78 | 74.30±20.04 | 199.45±22.42 |

Abbreviations: HYSHF: Huaiyangshan hemorrhagic fever, HFRS: hemorrhagic fever with renal syndrome.

-: not available.

Table S4-4 Dynamic profile of the CK in patients with HYSHF, HFRS, and undetermined infection.

| Course  (day) | HYSHF | HFRS | Undetermined infection |
| --- | --- | --- | --- |
|  | Mean ± SD | Mean ± SD | Mean ± SD |
| 1 | - | - | - |
| 2 | - | - | - |
| 3 | - | 21.00±0.00 | 138.00±0.00 |
| 4 | 144.33±129.85 | 214.86±293.62 | 116.83±90.54 |
| 5 | 453.00±398.76 | 97.57±129.24 | 143.75±137.79 |
| 6 | 603.75±702.33 | 262.86±328.35 | 223.00±163.36 |
| 7 | 1170.25±1120.45 | 154.50±238.99 | 489.50±574.96 |
| 8 | 1080.13±1109.62 | 105.87±108.07 | 213.00±265.61 |
| 9 | 640.93±706.24 | 190.00±143.94 | 123.67±75.82 |
| 10 | 948.17±1264.78 | 97.90±140.48 | 114.33±23.18 |
| 11 | 1064.00±1094.30 | 202.50±238.11 | 156.40±89.22 |
| 12 | 479.19±803.28 | 110.83±139.06 | 46.00±2.82 |
| 13 | 491.60±816.70 | 45.71±21.41 | 65.67±19.50 |
| 14 | 379.58±717.20 | 32.67±10.07 | 40.50±14.85 |
| 15 | 514.67±843.50 | 83.00±95.66 | 35.50±14.85 |

Abbreviations: HYSHF: Huaiyangshan hemorrhagic fever, HFRS: hemorrhagic fever with renal syndrome.

-: not available.

Table S4-5 Dynamic profile of the LDH in patients with HYSHF, HFRS, and undetermined infection.

| Course  (day) | HYSHF | HFRS | Undetermined infection |
| --- | --- | --- | --- |
|  | Mean ± SD | Mean ± SD | Mean ± SD |
| 1 | - | - | - |
| 2 | - | - | - |
| 3 | - | 450.00±0.00 | 214.00±0.00 |
| 4 | 376.00±206.83 | 549.14±285.12 | 287.50±109.46 |
| 5 | 856.50±650.94 | 475.14±252.21 | 315.00±224.69 |
| 6 | 1256.00±791.30 | 593.21±280.06 | 472.50±378.17 |
| 7 | 1555.75±963.99 | 568.67±278.16 | 627.00±366.66 |
| 8 | 1410.06±1020.08 | 558.80±378.49 | 642.33±311.34 |
| 9 | 1435.67±790.26 | 565.56±158.26 | 478.50±234.25 |
| 10 | 1359.00±796.01 | 458.50±185.37 | 539.33±203.30 |
| 11 | 1501.75±689.80 | 485.33±261.30 | 389.60±225.92 |
| 12 | 848.31±601.15 | 443.83±201.70 | 455.50±166.17 |
| 13 | 879.10±760.71 | 333.86±78.84 | 344.33±150.90 |
| 14 | 669.50±523.03 | 296.33±72.89 | 438.50±2.12 |
| 15 | 702.83±553.38 | 376.50±144.34 | 321.00±165.46 |

Abbreviations: HYSHF: Huaiyangshan hemorrhagic fever, HFRS: hemorrhagic fever with renal syndrome.

-: not available.

Table S4-6 Dynamic profile of the HBDH in patients with HYSHF, HFRS, and undetermined infection.

| Course  (day) | HYSHF | HFRS | Undetermined infection |
| --- | --- | --- | --- |
|  | Mean ± SD | Mean ± SD | Mean ± SD |
| 1 | - | - | - |
| 2 | - | - | - |
| 3 | - | 253.00±0.00 | 118.00±0.00 |
| 4 | 176.00±54.67 | 319.29±147.55 | 166.50±65.49 |
| 5 | 381.17±224.52 | 294.57±138.01 | 180.00±122.59 |
| 6 | 512.13±269.17 | 354.36±161.54 | 303.50±253.10 |
| 7 | 741.63±483.34 | 350.75±150.13 | 350.33±210.80 |
| 8 | 592.93±393.07 | 333.33±172.40 | 337.33±143.62 |
| 9 | 686.27±337.82 | 340.33±82.77 | 292.50±174.54 |
| 10 | 712.20±564.26 | 283.90±96.45 | 336.33±154.58 |
| 11 | 942.42±611.03 | 300.00±153.15 | 249.80±152.12 |
| 12 | 483.06±359.12 | 271.67±98.71 | 262.00±76.37 |
| 13 | 453.70±292.95 | 225.57±61.39 | 246.00±106.38 |
| 14 | 371.67±222.64 | 185.50±41.95 | 282.50±48.79 |
| 15 | 314.67±275.71 | 257.50±86.83 | 158.50±41.72 |

Abbreviations: HYSHF: Huaiyangshan hemorrhagic fever, HFRS: hemorrhagic fever with renal syndrome.

-: not available.

Table S4-7 Dynamic profile of the BUN in patients with HYSHF, HFRS, and undetermined infection.

| Course  (day) | HYSHF | HFRS | Undetermined infection |
| --- | --- | --- | --- |
|  | Mean ± SD | Mean ± SD | Mean ± SD |
| 1 | - | - | - |
| 2 | - | - | - |
| 3 | - | 10.38±2.08 | 5.53±0.00 |
| 4 | 4.68±1.29 | 15.72±6.39 | 5.18±1.61 |
| 5 | 5.30±2.86 | 20.58±6.59 | 4.44±1.32 |
| 6 | 5.74±4.52 | 18.17±8.72 | 9.97±11.40 |
| 7 | 6.03±4.96 | 23.29±11.53 | 20.84±20.45 |
| 8 | 6.81±3.26 | 21.95±11.28 | 26.84±24.80 |
| 9 | 5.69±2.57 | 26.64±13.33 | 23.46±25.07 |
| 10 | 5.18±2.47 | 20.90±12.94 | 26.49±25.63 |
| 11 | 6.11±3.16 | 17.58±11.15 | 19.87±22.42 |
| 12 | 5.06±2.21 | 18.47±9.79 | 5.52±1.90 |
| 13 | 3.95±1.39 | 10.00±7.16 | 11.94±15.66 |
| 14 | 5.42±2.87 | 11.47±9.71 | 16.70±18.15 |
| 15 | 5.63±4.11 | 11.23±6.93 | 22.20±23.47 |

Abbreviations: HYSHF: Huaiyangshan hemorrhagic fever, HFRS: hemorrhagic fever with renal syndrome.

-: not available.

Table S4-8 Dynamic profile of the CREA in patients with HYSHF, HFRS, and undetermined infection.

| Course  (day) | HYSHF | HFRS | Undetermined infection |
| --- | --- | --- | --- |
|  | Mean ± SD | Mean ± SD | Mean ± SD |
| 1 | - | - | - |
| 2 | - | - | - |
| 3 | - | 59.00±0.00 | 71.00±0.00 |
| 4 | 85.67±19.50 | 257.86±123.24 | 94.83±19.24 |
| 5 | 78.00±18.10 | 352.78±181.78 | 84.50±12.05 |
| 6 | 87.18±59.11 | 309.42±209.23 | 181.83±233.29 |
| 7 | 79.27±46.43 | 413.14±253.82 | 357.33±326.89 |
| 8 | 83.39±24.25 | 379.17±229.03 | 399.00±352.02 |
| 9 | 75.00±25.72 | 439.40±288.41 | 384.50±360.59 |
| 10 | 74.80±28.80 | 361.10±226.55 | 420.00±372.25 |
| 11 | 89.27±60.07 | 288.64±234.28 | 296.57±332.28 |
| 12 | 70.44±27.53 | 379.18±293.16 | 107.67±47.12 |
| 13 | 61.40±12.66 | 160.00±101.84 | 223.80±338.97 |
| 14 | 69.45±34.10 | 239.75±230.44 | 320.00±399.65 |
| 15 | 70.63±41.27 | 190.50±198.14 | 445.00±526.09 |

Abbreviations: HYSHF: Huaiyangshan hemorrhagic fever, HFRS: hemorrhagic fever with renal syndrome.

-: not available.
